# Characterization of the Soybean *GPAT* Gene Family Identifies *GmGPAT1* as a Key Protein in Salt Stress Tolerance

**DOI:** 10.3390/plants14182862

**Published:** 2025-09-13

**Authors:** Xin Li, Yunlong Li, Yan Sun, Sinan Li, Quan Cai, Shujun Li, Minghao Sun, Tao Yu, Xianglong Meng, Jianguo Zhang

**Affiliations:** Maize Research Institute of Heilongjiang Academy of Agricultural Sciences, Harbin 150086, China; maize_lee@163.com (X.L.); 13945699869@163.com (Y.L.); sunyan19850301@163.com (Y.S.); lee18686774002@126.com (S.L.); cq6539@163.com (Q.C.); lshj_750425@163.com (S.L.); sunminghao_yg@yeah.net (M.S.); yutaoweiwei@163.com (T.Y.); yjun00@126.com (X.M.)

**Keywords:** soybean, GPAT, salt stress, transgenic plants

## Abstract

Glycerol-3-phosphate acyltransferases (GPATs) catalyze the initial and rate-limiting step of glycerolipid biosynthesis, yet their contribution to salt tolerance in the soybean (*Glycine max* (L.) Merr.) plants remains largely uncharacterized. In this study, a total of 27 *GmGPAT* genes were identified, and their evolutionary relationships, chromosomal distribution, conserved motifs, and cis-regulatory elements were comprehensively analyzed. Through transcriptomic and qPCR analyses, many *GmGPATs* were found to be predominantly expressed in roots, with *GmGPAT1*, a plastid-targeted isoform, displaying the most rapid and pronounced transcriptional activation under salt stress. GFP-fusion experiments in transient expression assays confirmed plastid localization of *GmGPAT1*. Heterologous expression in *Escherichia coli* together with enzyme kinetics analyses validated its enzymatic function as a GPAT family member. The soybean hairy-root lines overexpressing *GmGPAT1* exhibited enhanced root elongation, increased biomass, and improved photosynthetic efficiency under 120 mM NaCl stress, while CRISPR/Cas9 knockout mutants showed pronounced growth inhibition. Physiological assays demonstrated that *GmGPAT1* overexpression mitigated oxidative damage by limiting reactive oxygen species (ROS) accumulation and lipid peroxidation, increasing antioxidant enzyme activities (CAT, SOD, POD), and elevating the ratios of AsA/DHA and GSH/GSSG. These changes contributed to redox homeostasis and improved Na^+^ extrusion capacity. A genome-wide association study (GWAS) involving 288 soybean accessions identified a single nucleotide polymorphism in the *GmGPAT1* promoter that was significantly correlated with salt tolerance, and the beneficial *Hap1* allele emerged as a promising molecular marker for breeding. Together, these analyses emphasize the status of *GmGPAT1* as a major regulator of salt stress adaptation through the coordinated modulation of lipid metabolism and redox balance, extend the functional annotation of the soybean GPAT family, and highlight new genetic resources that can be leveraged to enhance tolerance to salt stress in soybean cultivars.

## 1. Introduction

The soybean is the most widely cultivated oilseed crop globally, serving as a vital and renewable source of vegetable oil and protein for human nutrition, livestock feed, and various industrial applications. In higher plants, vegetable oil is primarily stored in seeds as triacylglycerols (TAGs), the biosynthesis of which involves a series of enzymatically regulated steps [1]. One of the key enzymes initiating this pathway is glycerol-3-phosphate acyltransferase (GPAT), which catalyzes the first acylation step by transferring a fatty acid (FA) from acyl-CoA to the hydroxyl group at the sn-1 position of glycerol-3-phosphate (G3P), resulting in the formation of lysophosphatidic acid (LPA) [2]. Next, lysophosphatidic acid acyltransferase (LPAAT) catalyzes the addition of a second FA to the sn-2 hydroxyl group of LPA, producing phosphatidic acid (PA). This intermediate is then dephosphorylated at the sn-3 position by phosphatidic acid phosphatase (PAP) to yield diacylglycerol (DAG). The final step in TAG assembly is catalyzed by either diacylglycerol acyltransferase (DGAT) or phospholipid/diacylglycerol acyltransferase (PDAT), which esterify a third FA derived from either acyl-CoA or phosphatidylcholine (PC) into the *sn*-*3* position of DAG, culminating in TAG formation [3,4,5].

As a critical enzyme necessary for TAG biosynthesis, GPAT (EC 2.3.1.15) plays essential roles not only in lipid metabolism but in broader physiological processes, such as plant growth, development, and responses to environmental stressors [6]. GPAT-encoding genes have been successfully isolated and characterized in various plants, including *Arabidopsis thaliana* [7], maize [8], rice [9], *Perilla frutescens* [10], Oilseed rape (*Brassica napus*) [11], *Helianthus annuus* [12], tomato [13], *Arachis hypogaea* [14], *Gossypium* [15], *Paeonia rockii* [16], and *Neochloris oleoabundans* [17]. These genes have been linked to various aspects of plant development and physiological function. In *Arabidopsis thaliana*, 10 *GPAT* isoforms have been identified and categorized based on their subcellular localization. Some are plastid-localized (e.g., *AT1G32200*), others reside in the mitochondria (*At1g06520, At1g02390, At4g01950*), while the remaining isoforms are found in the endoplasmic reticulum (*At1g01610*, *At3g11430*, *At2g38110*, *At5g06090*, *AT4G00400*, *AT5G60620*) [7]. These isoforms exhibit diverse biological roles. For example, *AtGPAT1* influences seed set in *Arabidopsis*, likely through the control of the biosynthesis of extracellular lipids [10]. *AtGPAT6* has been shown to play multiple roles in stamen development and fertility, such that mutants, in which its function has been disrupted, exhibit a pronounced seed yield reduction due to pollen abortion and impaired pollen wall formation [18]. In *Brassica napus*, *GPAT4* has been identified as a key regulator of reproductive organ and seed development. RNA interference (RNAi)-mediated suppression of *GPAT4* homologs in this species results in developmental abnormalities in reproductive tissues and a dysregulated seed set [19]. Conversely, overexpressing *BnGPAT* enhances seed oil accumulation in oilseed rape [11]. In *Lobosphaera incisa*, site-directed mutagenesis of LiGPAT has been reported to increase the phospholipid content in yeast [20]. Similarly, *AhGPAT9* influences seed oil levels in the peanut (*Arachis hypogaea*). In *Paeonia rockii*, *GPAT* is involved in the biosynthesis of alpha-linolenic acid (ALA), which is a key polyunsaturated fatty acid [21].

Beyond their roles in plant growth and lipid metabolism, GPATs are also integral to plant stress responses. Many studies have demonstrated their involvement in coping with adverse environmental conditions, such as high salinity [13] and cold exposure [22]. For instance, Sui et al. exposed tomato plants to 4 °C for various durations. Northern blotting revealed that the expression of *LeGPAT* was upregulated under low temperature conditions, peaking at 3 h and subsequently declining after 16 h [13]. By contrast, transgenic tomato plants with antisense suppression of the chloroplast-localized *LeGPAT* gene exhibited reduced heat stress damage [23]. Most *GhGPAT* genes were upregulated in the roots during moderate salt exposure, with several members exhibiting coordinated responses to both salt and cold stress [15,24]. The enhanced expression of GPAT genes has also been linked to improved salt tolerance in sweet sorghum (Sorghum bicolor M-81E), underscoring the involvement of membrane lipid modulation in salt-stress defense [25].

Despite these insights into how these genes shape stress responses in model systems and other crops, the roles of GPATs in the soybean under stress conditions remain largely unexplored. In this study, a systematic analysis of the *GPAT* gene family in the soybean was conducted, leading to the identification of 27 members together with the characterization of their gene structures, phylogenetic relationships, chromosomal locations, and regulatory elements. Subcellular localization predictions were supported by the transient expression of GFP-tagged GmGPAT proteins in *Arabidopsis* protoplasts, confirming their organelle-specific targeting profiles. Transcript abundance patterns across tissues and in response to abiotic stresses were assessed using qPCR and high-throughput transcriptomic datasets. Of the family members identified herein, GmGPAT1, a chloroplast-localized isoform, demonstrated the strongest transcriptional response to salt exposure and corresponded closely with enzymatic activity, indicating a potential primary role in salinity adaptation. The expression of *GmGPAT1* in *E. coli* confirmed its functional acyltransferase activity. Furthermore, transgenic soybean hairy roots overexpressing *GmGPAT1* exhibited improved tolerance to salt stress, characterized by elevated AsA/DHA and GSH/GSSG ratios, together with reduced ROS and lipid peroxidation levels. These findings suggest that GPATs contribute to stress resilience in soybeans, with *GmGPAT1* playing a key role in redox regulation and oxidative defense under high-salt conditions.

## 2. Materials and Methods

### 2.1. Plant Materials and Treatments

DN50 was planted in vermiculite and watered daily. After four days, the plants were transferred to a greenhouse with the following conditions: 16 h of light and 8 h of darkness at a white light intensity of 350 μmol/(m^2^·s), 600 ppm CO_2_, 80% relative humidity, and a temperature of 25 °C. Once the cotyledons emerged, intact seedlings were carefully moved to hydroponic boxes and, following the full expansion of the second trifoliate leaf, multiple abiotic stress treatments were applied, including various hormonal stress treatments.

### 2.2. Soybean GPAT Gene Family Identification

The complete soybean genome sequence was obtained from the Phytozome database (https://phytozome-next.jgi.doe.gov/ accessed on 10 September 2024). To identify the *GPAT* gene family members in the soybean, protein sequences of *Arabidopsis thaliana* GPATs (Table S1) were used as queries to search against the soybean genome using the BLASTP algorithm with a default E-value cutoff of 1 × 10^−5^. Redundant sequences were eliminated through self-BLAST comparison. Candidate genes were verified for the presence of the conserved PlsC acyltransferase domain (Pfam ID: PF01553) using the Pfam (http://pfam.sanger.ac.uk/search accessed on 10 September 2024) and SMART (http://smart.embl-heidelberg.de/ accessed on 10 September 2024) databases. Key gene features, including coding sequence length, chromosomal position, and predicted protein size, were obtained from the SoyBase database. Theoretical isoelectric points and molecular weights were computed with the ExPASy tool (http://expasy.org/ accessed on 10 September 2024). Predictions of subcellular localization were performed with TargetP 2.0 (http://www.cbs.dtu.dk/services/TargetP/ accessed on 15 September 2024) and CELLO 2.5 (http://cello.life.nctu.edu.tw/ accessed on 15 September 2024) [26].

### 2.3. GmGPAT Evolution, Gene Structure, and Synteny Analyses

To clarify evolutionary relationships, full-length GPAT protein sequences from *Glycine max* (*GmGPATs*), *Zea mays* (*ZmGPATs*), *Oryza sativa* (*OsGPATs*), *Sorghum bicolor* (*SbGPATs*), and *A. thaliana* (*AtGPATs*) were aligned and used to construct a phylogenetic tree via the neighbor-joining method in MEGA 5.0 using the default settings [27]. The exon–intron organization of each gene was determined using the Gene Structure Display Server (GSDS) by aligning the coding sequences with the corresponding genomic DNA. Syntenic relationships among the GPAT genes from *G. max*, *Z. mays*, *A. thaliana*, *O. sativa*, and *S. bicolor* were identified using the Plant Genome Duplication Database (PGDD8) [28]. The gene identifiers and additional details are listed in Supplementary Table S1. Conserved motifs among the soybean GPAT proteins were analyzed using the MEME suite (http://meme-suite.org/ accessed on 19 September 2024) [29], using the default parameters, aside from the identification of up to 20 motifs.

### 2.4. GmGPAT Promoter Analysis

To explore the transcriptional regulatory mechanisms, 2 kb sequences upstream of the ATG start codon for each *GmGPAT* gene were retrieved from the soybean genome database. These promoter regions were analyzed for cis-regulatory elements using the PlantCARE database (https://bioinformatics.psb.ugent.be/webtools/plantcare/html/ accessed on 19 September 2024). The identified elements were visualized using IBS 2.0 [30].

### 2.5. Subcellular Localization

The full-length coding sequences of *GmGPAT1* (*Glyma.01G014200*) and *GmGPAT4* were amplified from root tissues of the soybean cultivar DN50, provided by the Soybean Breeding Research Center at Northeast Agricultural University (Harbin, China). Amplification was carried out via RT-PCR with high-fidelity KOD-Plus DNA polymerase (TOYOBO, Osaka, Japan). The resulting PCR products were cloned into the *pBI121* binary vector, which contains a constitutive CaMV35S promoter and a green fluorescent protein (GFP) reporter gene. The primer sequences used for gene cloning are listed in Supplementary Table S2. The recombinant constructs (*pBI121-GmGPAT1/GFP* and *pBI121-GmGPAT4/GFP*), along with an empty vector control, were transiently expressed in *A. thaliana* mesophyll protoplasts. The protoplasts were prepared from 14-day-old seedlings grown under low-light conditions to minimize chlorophyll autofluorescence. Subcellular localization was assessed 16 h post-transfection using a polyethylene glycol (PEG)-mediated transformation protocol [31].

### 2.6. qPCR

Total RNA was extracted from the soybean samples using the Quick Total RNA Isolation Kit (Beijing Huayueyang Biotechnology Co., Ltd., Beijing, China), and its purity and integrity were confirmed via spectrophotometry and 1% agarose gel electrophoresis. First-strand cDNA was synthesized using PrimeScript™ RT Master Mix (Takara Bio Inc., Dalian, China) and diluted to a working concentration of 500 ng/μL. The qPCR assay was performed using SYBR Green I Master Mix (Takara Bio Inc.) on a Roche LightCycler^®^ 96 instrument [32]. Each 20 μL reaction contained 10 μL of 2× SYBR Green PCR Mix, 0.5 μL of each gene-specific primer, and diluted cDNA. Thermal cycling conditions included 95 °C for 30 s, followed by 45 cycles of 95 °C for 15 s and annealing at 55–60 °C. The relative expression of *GmGPAT* genes was normalized to two internal controls: *GmGAPDH* (GenBank accession: DQ355800) and *GmACTIN* (GenBank accession: AF049106) [33]. The sample size (*n* = 9) includes 3 biological replicates and 3 technical replicates. Transcript abundance was quantified using the 2^−ΔΔCt^ method. The primers are listed in Supplementary Table S2.

### 2.7. Recombinant Protein Expression and Enzyme Kinetic Activity Assays

The coding sequence of *GmGPAT1* was inserted into the *pET28a*(+) expression vector through the use of *HindIII* and *BamHI* restriction sites. The recombinant plasmid (*pET28a–GmGPAT1*) was transformed into *E. coli* Rosetta cells for protein expression. Following confirmation by sequencing, the transformed cells were cultured in an LB medium and induced with 1 mmol L^−1^ IPTG at 37 °C for 4 h. His-tagged proteins were purified using Ni-NTA affinity chromatography and validated via Western blotting using anti-His antibodies. The GPAT enzymatic activity was determined in a reaction mixture containing 50 nmol DTNB, 1 μmol glycerol-3-phosphate, and 20–320 μg of membrane protein in 0.1 M phosphate buffer (pH 7.2), with a final volume of 400 μL. After an initial delay (30–60 s, depending on the enzyme concentration), absorbance at 405 nm was recorded for 4 min under conditions that ensured a linear reaction rate. The enzyme activity was calculated using a molar extinction coefficient of 13,600.

### 2.8. Agrobacterium-Mediated Overexpression and Knockout of GmGPAT1

To investigate the functional role of *GmGPAT1*, overexpression and knockout constructs were generated. The coding sequence of *GmGPAT1* was cloned into the *pSOY1* vector under the control of a CaMV35S promoter, creating the overexpression construct pSOY1–GmGPAT1. For gene knockout, two sgRNAs (Table S4) targeting the *GmGPAT1* coding region were designed and introduced into a CRISPR/Cas9 system. *Agrobacterium rhizogenes* strain K599 harboring the recombinant vectors (*pSOY1–GmGPAT1* or *pGES401–GmGPAT1*) was used to transform soybean hypocotyls from the DN50 cultivar via electroporation, following the protocols described in a prior report [34]. Hairy root induction and transformation efficiency were validated through PCR and enzyme activity assays, and non-transformed roots were excised. Mutations in the CRISPR-edited lines were characterized using Hi-TOM sequencing. Genomic DNA was extracted from the plant tissues and then amplified using specific primers targeting the edited regions. The amplified fragments were subsequently sequenced (Table S4). At least 10–20 independent transgenic hairy root lines were analyzed to assess phenotypic and physiological responses. Salt stress experiments were conducted by treating plants with 0 or 120 mM NaCl for 5 days. Root length and root and leaf biomass were recorded, and photosynthetic efficiency was assessed using a Plant Explorer PSII HS chlorophyll fluorescence imager (PhenoVation Life Sciences, Wageningen, Netherlands).

### 2.9. Reactive Oxygen and Antioxidant Enzyme Analyses

Biochemical assays were conducted on transgenic hairy root samples collected after 5 days of treatment with 0 or 120 mM NaCl. Tissues were flash-frozen in liquid nitrogen and homogenized, followed by immediate extraction using appropriate buffers. Hydrogen peroxide (H_2_O_2_) and superoxide (O_2_•^−^) levels were quantified spectrophotometrically, as described in prior publications [35,36]. For antioxidant analysis, 0.5 g of root tissue was extracted in 15 mL of a buffer containing 50 mmol L^−1^ K_2_HPO_4_–KH_2_PO_4_ (pH 7.0), 1.5 mmol L^−1^ EDTA, 1% (*w*/*v*) polyvinylpyrrolidone (PVP), and 0.5 mmol L^−1^ ascorbate. The resulting supernatants were assayed for the activities of superoxide dismutase (SOD), peroxidase (POD), and ascorbate peroxidase (APX) using established protocols [37,38].

### 2.10. Haplotype Analyses of GmGPAT1

Haplotype variation in *GmGPAT1* was examined in a panel of 288 Chinese soybean accessions selected for their genetic diversity and range of salt tolerance indices (STIs). Plants were grown hydroponically to the V3 developmental stage and subjected to 120 mM NaCl for 5 days. Phenotypic traits, including plant height, root length, and fresh weight of both aerial and root tissues, were recorded. Salt tolerance indices were calculated using principal component analysis, membership function analysis, and weighting of composite indices [39,40,41]. Candidate gene sequences were retrieved from the Phytozome database, and SNPs were identified by aligning *GmGPAT1* sequences from the resequencing data of the 288 accessions (unpublished). Local BLAST (Basic Local Alignment Search Tool) analyses were used to extract SNPs, and low-quality haplotypes (frequency <5%) were excluded. DnaSP v5.0 [42] was used to evaluate the haplotype distributions. Phenotypic differences among the haplotypes were statistically analyzed via ANOVAs with Duncan’s multiple range test in SPSS.

### 2.11. Statistical Analysis

All experiments were conducted in triplicate, with each biological replicate comprising an independent experimental unit. Data are expressed as mean ± standard deviation (SD). Statistical significance was determined using Fisher’s least significant difference (LSD) test, with a confidence threshold set at *p* < 0.05, using SPSS 27.0.

## 3. Results

### 3.1. Soybean GPAT Identification

To identify *GPAT* genes in the soybean, a BLAST search was conducted against the Phytozome v12.1 database using the conserved domains from ten *Arabidopsis* GPAT proteins as queries [43,44,45]. This analysis yielded 27 putative *GPAT* genes in the soybean, designated *GmGPAT1* through *GmGPAT27* (Table S1). The predicted protein products ranged from 374 to 557 amino acids in length. Their molecular weights ranged from 42.82 to 63.20 kDa, while their isoelectric points ranged from 8.33 to 9.81 (Table S1).

**Figure 1:**
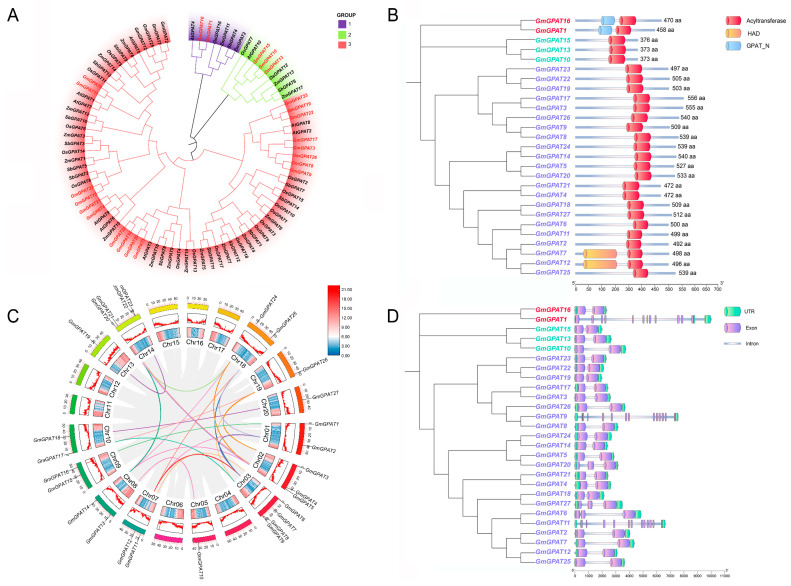
Phylogenetic relationships, conserved domains, synteny, and gene structure of the *GmGPAT* family in the soybean. (**A**) A phylogenetic tree was constructed using GPAT protein sequences from *Glycine max, Arabidopsis thaliana, Zea mays, Oryza sativa,* and *Sorghum bicolor*. Species are represented by distinct colors to reflect taxonomic diversity. (**B**) Conserved protein domains in the soybean GPATs were analyzed, with the Acyltransferase, GPAT_N, and HAD domains shown in red, blue, and yellow boxes, respectively. (**C**) Intragenomic synteny analysis of the *GmGPAT* genes reveals duplicated segments within the soybean genome. Colored lines link gene pairs based on sequence similarity, with line color corresponding to log_2_ expression levels. Red denotes higher expression, and blue denotes lower expression; chromosomes are illustrated as color-coded bars. (**D**) The exon–intron architecture of the *GmGPAT* genes, with green boxes indicating untranslated regions (UTRs), purple boxes representing exons, and blue lines indicating introns.

Phylogenetic relationships were inferred by aligning the full-length protein sequences of the soybean *GPAT* candidates with those from *S. bicolor*, *Z. mays*, *A. thaliana*, *G. max*, and *O. sativa*. The resulting tree revealed three major clades (Figure 1A). Clade I comprised *GmGPAT1*, *GmGPAT16*, and *Arabidopsis AtGPAT4*. Clade II included *GmGPAT10*, *GmGPAT13*, *GmGPAT15*, along with *AtGPAT10*. Clade III, the largest group, consisted of 22 GmGPAT genes (*GmGPAT2–9*, *11*, *12*, *14*, *17–27*) and 8 Arabidopsis members (*AtGPAT1–3*, *AtGPAT5–9*).

### 3.2. GmGPAT Synteny and Gene Structure Analyses

**Figure 2:**
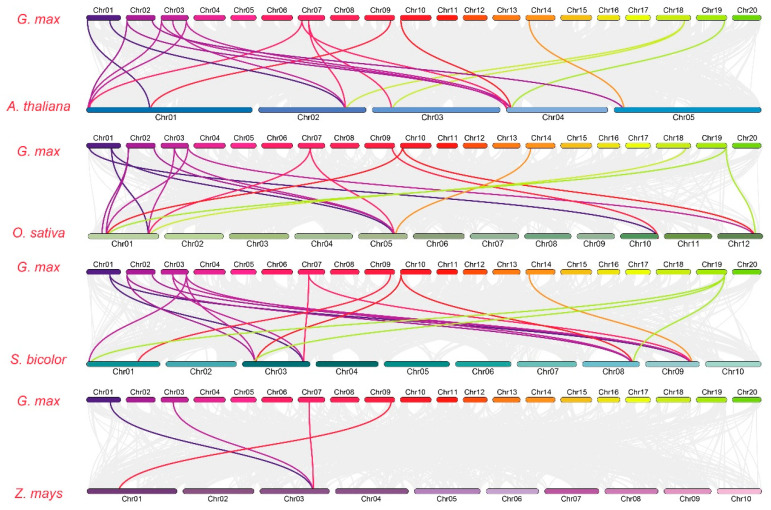
Comparative synteny of the soybean *GPAT* genes with those in other plant species. Syntenic relationships between the soybean GPAT genes and homologous genes from *A. thaliana*, *O. sativa*, *S. bicolor*, and *Z. mays* are shown. Colored lines depict chromosomes, and connecting curves indicate conserved collinear regions.

**Figure 3:**
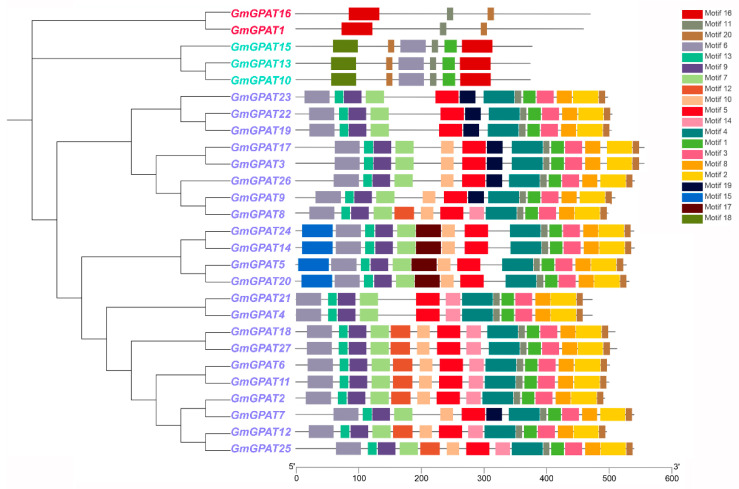
Conserved motif and gene structure analyses of the soybean *GPAT* family genes. The *GPAT* genes are grouped into three subfamilies, each highlighted in a different color. Colored blocks on the right represent the 20 conserved motifs identified in the protein sequences.

A domain architecture analysis revealed that both *GmGPAT1* and *GmGPAT16* harbor the GPAT_N domain, whereas *GmGPAT10, GmGPAT13*, and *GmGPAT15* share a similar protein length and consistent arrangement of the Acyltransferase domain. The remaining GmGPAT proteins also show conserved Acyltransferase domain positioning and comparable sequence lengths (Figure 1B). Chromosomal mapping indicated that the *GmGPAT* genes are distributed across 13 of the 20 soybean chromosomes, with 22 pairs exhibiting collinearity (Figure 1C). A comparative synteny analysis revealed 14 orthologous gene pairs between the soybean and *A. thaliana*, 18 with *O. sativa*, 16 with *S. bicolor*, and 4 with *Z. mays* (Figure 2; Table S3), providing insight into the evolutionary conservation and divergence of the *GPAT* gene family. A gene structure analysis indicated a conserved exon–intron organization within the subfamilies. While *GmGPAT1*, *GmGPAT9*, and *GmGPAT11* were each found to contain 12 exons, most of the remaining members harbored just 2 exons (Figure 1D). A conserved motif analysis using MEME identified 20 distinct sequence motifs across the GmGPAT proteins (Figure 3). Motifs 11, 16, and 20 were uniquely present in both GmGPAT1 and GmGPAT6, while Motif 6 was absent in only these two family members. Notably, GmGPAT10, GmGPAT13, and GmGPAT15 shared six motifs (Motifs 1, 6, 11, 16, 18, and 20), reflecting the conserved nature of functional domains within the subfamilies.

### 3.3. GmGPAT Promoter Regulatory Element Analysis

**Figure 4:**
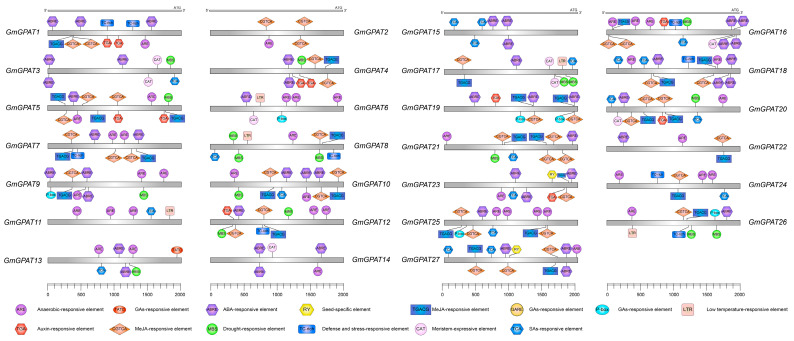
*Cis*-acting Elements within 2000 bp of the Promoter. Candidate *cis*-acting elements located within 2000 bp upstream of *GmGPAT* genes were identified.

To explore the potential regulatory mechanisms involved in the saline–alkali stress responses, the 2000 bp upstream promoter regions of all *GmGPAT* genes were analyzed for *cis*-acting elements (Figure 4). The hormone-responsive elements are widely present among family members, including ABA-responsive elements (ABRE), MeJA-responsive motifs (TGACG and CGTCA), salicylic acid-responsive motifs (TCA), and gibberellin-responsive elements (P-box, GARE, TATC-box), suggesting that these genes play important roles in the physiological regulation of plants. ABREs were present in 23 of 27 *GmGPAT* promoters, absent only in *GmGPAT2*, *8*, *11*, and *24*. MeJA-responsive elements were detected in *GmGPAT1*, *2*, *4*, *5*, *7*, *8*, *10*, *12*, and *16–27*. Elements linked to abiotic stress responses, particularly cold-responsive motifs, were found in GmGPAT6, 8, 11, 17, and 26, indicating that these genes may be involved in the adaptation of plants to salt–alkali and low-temperature stress. In addition, growth- and development-related elements, such as RY and CAT, were identified; RY motifs were present in GmGPAT23 and 27, while CAT elements were found in GmGPAT3, 6, 14, 16, 17, and 20. These data suggest that the *GmGPAT* expression patterns are tightly regulated and potentially involved not only in stress adaptation but in key roles during plant growth and developmental processes. These analyses suggest that the expression patterns of the *GmGPAT* genes are potentially regulated by complex interactions among multiple signaling pathways, providing diverse avenues for future research.

### 3.4. GmGPAT Subcellular Localization Analysis

**Figure 5:**
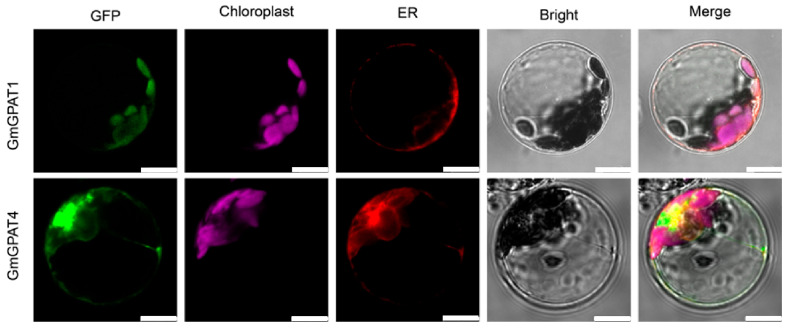
Analysis of the subcellular *GmGPAT* localization. Fusion proteins of GmGPAT1 and GmGPAT4 with GFP were transiently expressed in *Arabidopsis thaliana* mesophyll protoplasts. Confocal microscopy images reveal their subcellular distribution. Scale bars = 10 μm.

To experimentally determine the subcellular localization of selected GmGPAT proteins, *GmGPAT1* and *GmGPAT4* were fused to enhanced GFP and transiently expressed in *A. thaliana* mesophyll protoplasts. Confocal microscopy revealed that the GFP signal from GmGPAT1 was co-localized with chlorophyll autofluorescence, indicating chloroplast localization. By contrast, GmGPAT4–GFP fluorescence overlapped with an endoplasmic reticulum (ER) marker (Figure 5). These localization patterns are consistent with computational predictions and the known distributions of the Arabidopsis orthologs, suggesting conserved roles in subcellular compartmentalization.

### 3.5. GmGPAT Tissue-Specific Expression Pattern Analysis

**Figure 6:**
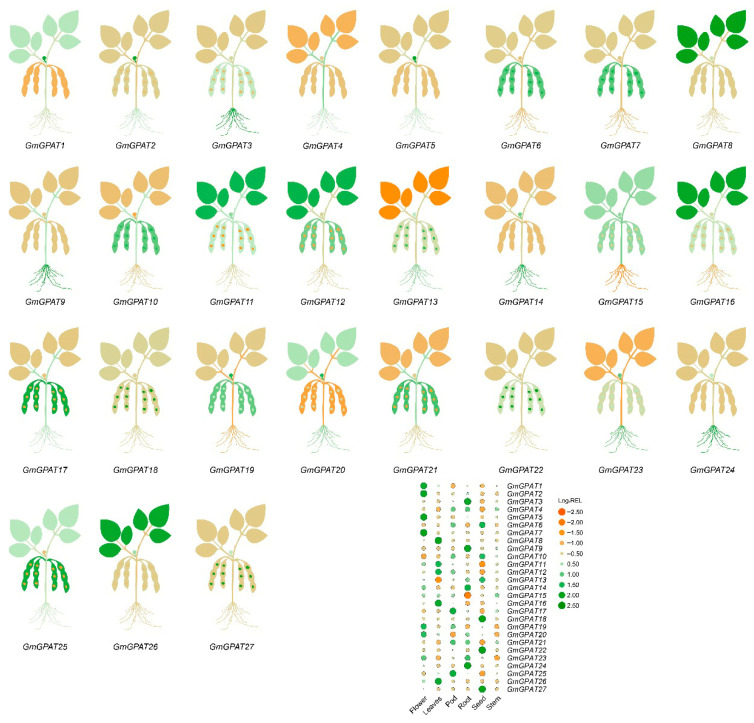
Patterns of *GmGPAT* gene expression across soybean tissues. Transcript abundance of *GmGPAT* genes was retrieved from the Phytozome database and visualized using a heatmap generated with TBtools (TBtools v2.056). All log_2_ expression values are color-coded, with green indicating low expression and orange representing high expression levels.

Expression profiling across various soybean tissues revealed that *GmGPAT* transcripts are detectable in flowers, leaves, nodules, pods, roots, seeds, shoot apices, and stems (Figure 6). An analysis shows that the expression levels varied significantly across tissues, with roots and leaves generally exhibiting the highest transcript abundance. This variation suggests their crucial roles in root development and environmental adaptation. Specifically, *GmGPAT1*, *GmGPAT2*, and *GmGPAT5* were found to have the highest expression levels in flowers, highlighting their potential significance during flower development. By contrast, *GmGPAT17* exhibited the highest expression in pods, indicating its possible involvement in pod maturation and seed development. Additionally, *GmGPAT6*, *GmGPAT10*, *GmGPAT13*, *GmGPAT18*, *GmGPAT22*, and *GmGPAT27* demonstrated the highest expression in seeds, suggesting critical roles in seed formation and viability. These results provide valuable insights into the functional diversity of the *GmGPAT* genes and underscore their importance in soybean tissue differentiation and developmental processes.

### 3.6. Analysis of GmGPAT Expression Patterns Under Different Stresses

**Figure 7:**
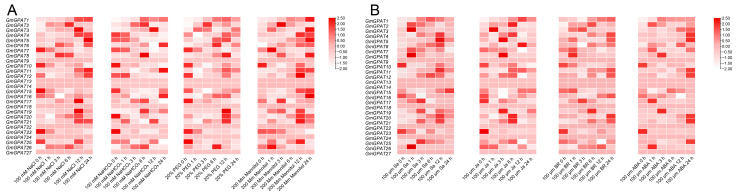
*GmGPAT* expression patterns under abiotic stress and hormonal treatments. Expression profiles of the *GmGPAT* genes in soybean leaves were monitored at 0, 1, 3, 6, 12, and 24 h following treatment with 150 mmol·L^−1^ NaCl, 100 mmol·L^−1^ NaHCO_3_, 20% PEG, or 200 mmol·L^−1^ mannitol (**A**), as well as exposure to 100 μmol·L^−1^ of JA, ABA, BR, or SA hormones (**B**). Water was used as a control. Heatmaps display log_2_ expression levels, where white signifies low transcript levels and red indicates high abundance.

To assess *GmGPAT* expression under abiotic stress conditions, soybean seedlings were subjected to osmotic stress (20% PEG or 200 mM mannitol), salt stress (150 mM NaCl), and alkaline conditions (100 mM NaHCO_3_) (Figure 7A). Salt stress induced the robust expression of multiple *GmGPAT* genes, with *GmGPAT1* showing the strongest response, peaking at 24 h post-treatment. Under alkaline conditions, GmGPAT1 displayed a biphasic expression pattern, with peaks at 6 h and 24 h. GmGPAT3 showed maximal induction at 12 h following both salt and alkali stress, suggesting a role in mid-to-late stress responses. Osmotic stress had a less pronounced effect overall, though *GmGPAT1*, *GmGPAT2*, and *GmGPAT4* exhibited moderate upregulation, especially at 6–12 h after PEG exposure. In light of the regulatory functions of plant hormones in stress signaling [46], we also evaluated the transcript levels of 17 *GmGPAT* genes in response to the exogenous application of ABA, SA, BR, and MeJA (Figure 7B). Most *GmGPATs* were significantly upregulated following ABA and BR treatments. *GmGPAT4*, *6*, *11*, and *12* showed the greatest responsiveness to ABA, while *GmGPAT16*, *17*, *19*, and *21* were particularly responsive to BR. Many *GmGPATs* responded to all four hormones, indicating their involvement in multiple hormone-mediated stress signaling pathways.

### 3.7. GmGPAT1 Overexpression Enhances Salt Tolerance

**Figure 8:**
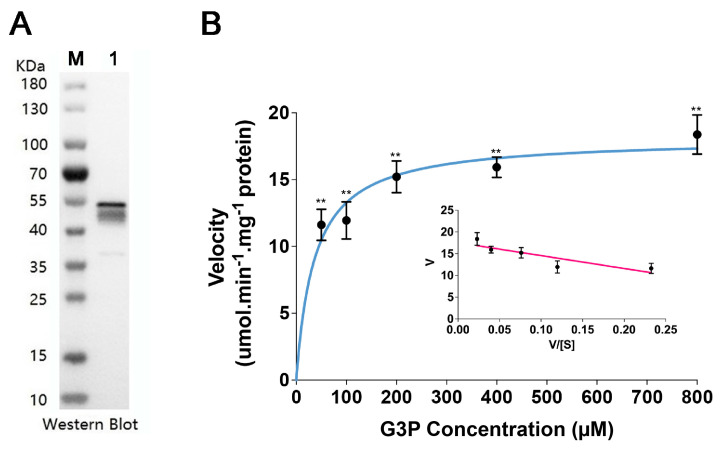
Expression and enzymatic characterization of recombinant soybean GmGPAT1. (**A**) Western blot analysis of purified GmGPAT1 fusion protein expression (Lane 1). Lane M, molecular weight markers. Fusion protein MW: 51.78 kDa. (**B**) The enzymatic activity of recombinant GmGPAT1 as quantified by assessing its catalytic efficiency in response to varying glycerol-3-phosphate (G3P) concentrations. Data are shown as mean ± standard deviation from three biological replicates, with each biological replicate having three technical replicates. ** *p* < 0.01.

To investigate the functional role of *GmGPAT1* in conferring salt stress resistance, we expressed a His-tagged version of the gene in *E. coli* and purified the recombinant protein for enzymatic analysis. SDS-PAGE confirmed that the molecular mass of the fusion protein corresponded to the predicted size (Figure 8A). GPATs catalyze the esterification of glycerol-3-phosphate (G3P) at the sn-1 position, generating lysophosphatidic acid (LPA). As such, the enzyme kinetics of the purified recombinant GmGPAT1 were assessed using G3P as a substrate. Based on the Eadie–Hofstee plots, the apparent Km for G3P was estimated to be 36.47 mmol L^−1^, while the maximum reaction velocity (Vmax) was determined to be 18.12 µmol min^−1^ mg^−1^ of protein (Figure 8B).

**Figure 9:**
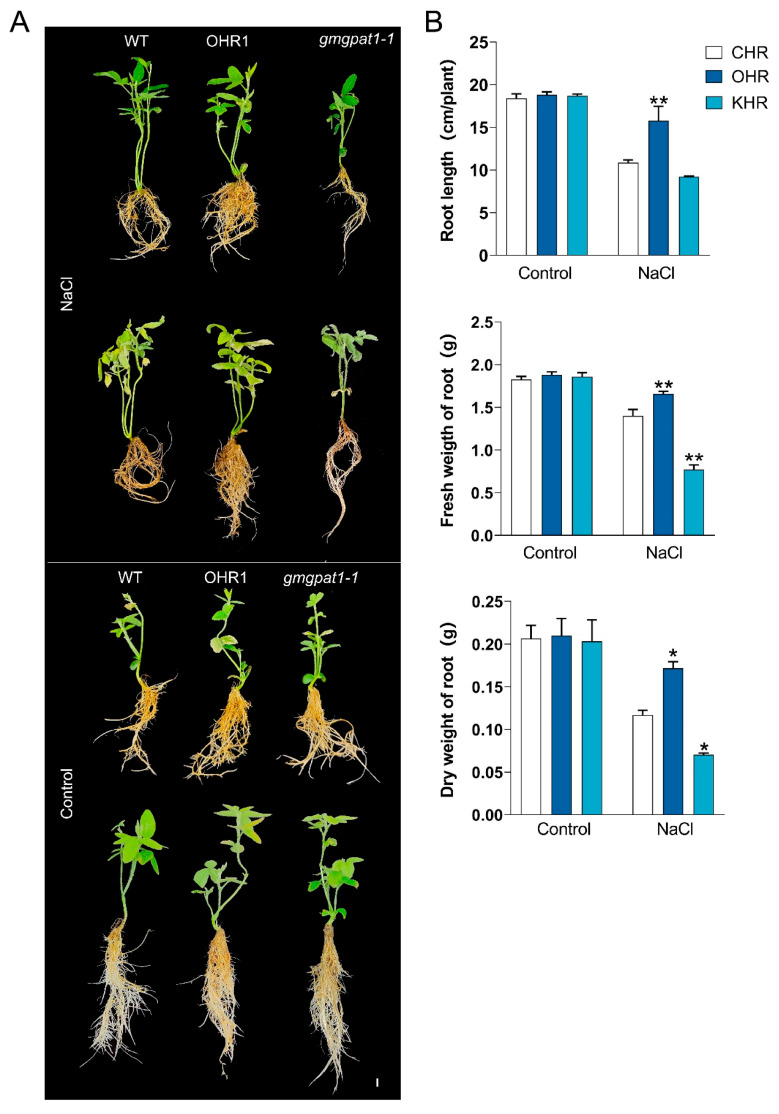
*GmGPAT1* overexpression enhances the saline–alkali stress tolerance in soybeans. (**A**) Phenotypic comparison of soybean plants with GmGPAT1-overexpressing hairy roots (OHR), GmGPAT1-knockout hairy roots (KHR), and control hairy roots (CHR) after exposure to 120 mM NaCl for 5 days. Scale bars = 1 cm. (**B**) Measurements of root length, fresh biomass, and dry biomass in response to stress treatment. The sample size (n = 9) includes three biological replicates and three technical replicates. * *p* < 0.05, ** *p* < 0.01; Student’s t-test compared to CHR controls.

To explore the in vivo role of *GmGPAT1*, transgenic soybean hairy roots were engineered via *Agrobacterium rhizogenes* transformation. Ten independent overexpression lines (GmGPAT1-OHR) were confirmed via PCR (Figure S1), and two knockout lines (GmGPAT1-KHR) containing premature stop codons were identified using Hi-TOM sequencing (Table S4). To evaluate their response to salt stress, three-week-old CHR (control), GmGPAT1-OHR, and GmGPAT1-KHR plants were treated with 120 mM NaCl for five days. The GmGPAT1-KHR plants exhibited pronounced growth retardation and visible shrinkage (Figure 9A), whereas the GmGPAT1-OHR lines demonstrated significantly enhanced salt tolerance, evidenced by increased root length and higher root fresh and dry biomass (Figure 9B).

Photosystem II functionality was also affected by salt stress, as revealed by chlorophyll fluorescence measurements. The maximum quantum efficiency of PSII photochemistry (Fv/Fm), minimum fluorescence in the dark-adapted state (F_0_), chlorophyll index (Chl-index), and anthocyanin index (Ari-index) were all diminished in GmGPAT1-KHR under salt stress conditions, whereas these parameters improved in the *GmGPAT1*-OHR lines relative to CHR (Figure S2). These results suggest that overexpression of *GmGPAT1* stabilizes photosynthetic performance under high-salt conditions, whereas its loss compromises photosystem efficiency.

### 3.8. GmGPAT1 Alleviates Oxidative Stress and Maintains Redox Homeostasis Under Salt Stress

**Figure 10:**
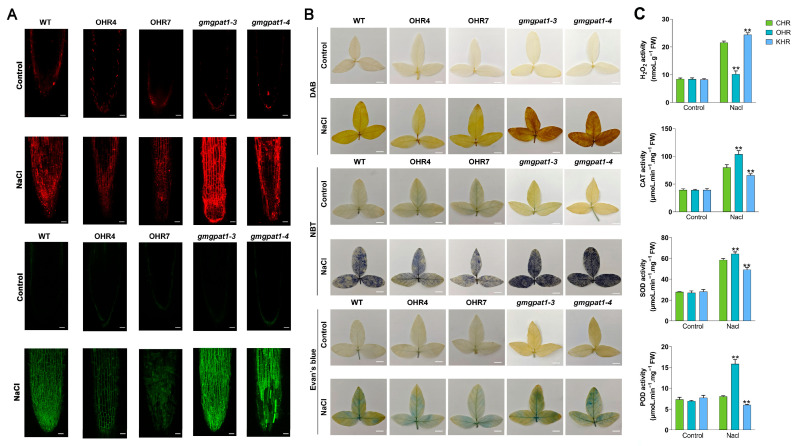
GmGPAT1 limits sodium ion accumulation, oxidative stress, and cell viability under salt stress. (**A**) Propidium Iodide (PI) staining and CoroNa^TM^ Green staining of the GmGPAT1-OHR, GmGPAT1-KHR, and CHR lines were conducted following NaCl exposure (0 or 120 mM). Scale bars = 100 μm. (**B**) DAB staining was used to detect H_2_O_2_ levels, while O^2-^ was detected through NBT staining, and cell death was assessed via Evan’s blue staining. Scale bars = 1 cm. (**C**) GmGPAT1 contributes to redox homeostasis under salt stress by modulating ROS levels. The sample size (n = 9) includes three biological replicates and three technical replicates. ** *p* < 0.01; Student’s t-test compared to CHR controls.

A hallmark of plant responses to abiotic stress is the accumulation of ROS, including O_2_^−^ and H_2_O_2_ [47]. To evaluate cell membrane integrity, PI staining, which fluoresces red upon binding to nuclear DNA but only penetrates cells with compromised membranes, was conducted. Under non-stressed conditions, PI fluorescence in all genotypes was minimal and indistinguishable. Following NaCl treatment (120 mM), the GmGPAT1-KHR roots showed the most intense PI staining, indicative of extensive membrane damage, while GmGPAT1-OHR roots exhibited the least staining, suggesting superior membrane stability under stress. Sodium ion accumulation was monitored using CoroNa™ Green, a fluorescent indicator that binds intracellular Na^+^. Without stress, all lines showed comparably weak staining (Figure 10A). After salt exposure, the GmGPAT1-KHR roots displayed the strongest fluorescence, reflecting elevated intracellular Na^+^ content. By contrast, the GmGPAT1-OHR roots exhibited the weakest signal, suggesting that *GmGPAT1* overexpression may facilitate sodium ion efflux, thereby enhancing osmotic stress tolerance.

ROS levels were further investigated through NBT and DAB staining strategies, which were respectively used to detect O_2_^−^ and H_2_O_2_ [48,49]. Under normal conditions, all plants exhibited faint and comparable staining. However, salt-stressed leaves from the GmGPAT1-KHR lines developed intense blue and brown pigmentation, reflecting elevated ROS accumulation, while the GmGPAT1-OHR leaves remained lightly stained (Figure 10B). Additionally, Evans blue staining, a marker for cell death, was employed to assess tissue viability [50]. Prior to stress treatment, minimal staining was observed across all lines. Following NaCl treatment, the GmGPAT1-KHR leaves displayed the most pronounced staining, indicative of significant cellular damage. By contrast, the GmGPAT1-OHR tissues exhibited reduced Evans blue uptake, suggesting a protective effect against salt-induced cytotoxicity. Collectively, these results highlight a crucial role for GmGPAT1 in enhancing salt stress resilience by modulating ROS scavenging systems, preserving membrane integrity, and limiting sodium accumulation. Overexpression of *GmGPAT1* thus contributes to improved cellular homeostasis and mitigates oxidative stress-related damage under saline conditions. To assess the involvement of GmGPAT1 in oxidative stress regulation, we measured H_2_O_2_ levels in the transgenic and the control lines. Notably, the GmGPAT1-OHR plants exhibited significantly reduced H_2_O_2_ accumulation compared to CHR, while the GmGPAT1-KHR plants accumulated elevated levels (Figure 10C), implicating GmGPAT1 in ROS detoxification. Key antioxidant enzymes, including catalase (CAT), peroxidase (POD), and superoxide dismutase (SOD), facilitate ROS scavenging in plant cells. Under salt stress, the activities of these enzymes were markedly enhanced in the GmGPAT1-OHR lines and suppressed in GmGPAT1-KHR, relative to control plants (Figure 10C). These findings suggest that GmGPAT1, which likely localizes to chloroplasts, contributes to maintaining cellular redox balance and mitigating oxidative damage during salt stress.

### 3.9. GmGPAT1 Haplotype Analysis

**Figure 11:**
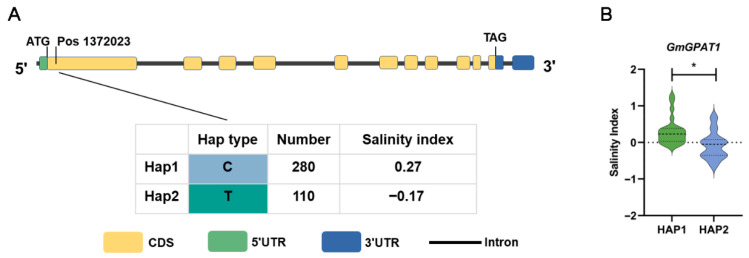
Haplotype analysis of *GmGPAT1* associated with salt tolerance in the soybean. (**A**) A haplotype analysis was conducted using 288 soybean accessions. Significant SNPs are mapped to the structural regions of the *GmGPAT1* gene. (**B**) A comparative analysis of stress tolerance indices (STI) among different haplotypes. * *p* < 0.05.

To explore natural allelic variation of the *GmGPAT1* gene across the diverse soybean germplasm, a haplotype analysis was conducted using 288 soybean accessions. This analysis focused on polymorphisms located within the gene’s coding and regulatory regions. Specifically, two non-synonymous SNPs in the coding sequence and eight significant SNPs within the promoter region were used to define genetic variation (Figure 11). Based on these variants, a total of 50 distinct haplotypes of *GmGPAT1* were identified, among which only Hap-1 and Hap-2 had frequencies greater than 0.05, classifying them as superior haplotypes. Phenotypic comparisons of salt stress tolerance among accessions carrying different haplotypes revealed that plants possessing *Hap-1* consistently exhibited a significantly higher stress tolerance index (STI) than those carrying *Hap-2* (Figure 11). These results suggest that specific natural variants within the *GmGPAT1* promoter region play a critical role in regulating gene function under saline conditions. The superior performance of *Hap-1* highlights its potential utility as a favorable allele for breeding salt-tolerant soybean cultivars. This further indicates that the regulatory mechanisms of different haplotypes vary from one another.

## 4. Discussion

The soybean is a globally important agricultural crop, providing a major source of plant-derived protein and oil for human consumption. However, soybean productivity is severely constrained by soil salinization, which is increasingly prevalent in many agricultural regions worldwide [51]. GPATs have been firmly established as key mediators of plant development and environmental stress adaptation [52,53,54]. Despite studies of GPATs in *A. thaliana* [7], maize [8], rice [9], and *Perilla frutescens* [10], the roles of these genes in the soybean remain poorly understood.

In the present study, we systematically identified and characterized 27 *GmGPAT* genes (*GmGPAT1–27*) in the soybean (Table S1). Consistent with previous findings in other species [8,55], these genes were categorized into three major subgroups based on predicted subcellular localization: chloroplast-targeted (GmGPAT1, 16), mitochondrial (GmGPAT10, 13, 15), and endoplasmic reticulum-localized isoforms (GmGPAT2–9, 11–12, 14, 17–27). A comparative domain analysis revealed that members of the same phylogenetic subgroup shared conserved motifs, including the GPAT_N domain in GmGPAT1, suggesting potential functional similarities among group members that warrant further validation.

Gene duplication events are known to contribute significantly to the expansion and diversification of gene families in plants. In *G. max*, we identified 33 segmental duplication events involving *GmGPAT* genes (Figure 2), supporting the conclusion that segmental rather than tandem duplications have primarily driven the evolutionary expansion of this gene family. These findings are in agreement with prior reports in *Gossypium barbadense*, where a similar duplication pattern was observed [15]. A collinearity analysis across multiple species revealed the strongest syntenic relationships between the soybean *GPAT* genes and their orthologs in *A. thaliana*, followed by moderate collinearity with rice (*OsGPAT*) and sorghum (*SbGPAT*) genes. The weakest synteny was noted with maize (*ZmGPAT*) (Figure 6), reflecting divergent evolutionary trajectories of this *GPAT* gene family across different plant lineages.

A promoter analyses further demonstrated that various *GmGPAT* genes contain a diverse array of *cis*-acting regulatory elements related to hormonal signaling, abiotic stress responses, and developmental processes (Figure 3). These include ARE, ABRE, and LTR motifs, which are typically associated with responses to oxidative stress, abscisic acid signaling, and low-temperature stress, respectively. Such promoter diversity implies that *GmGPAT* genes are intricately involved in the coordination of hormonal and stress-related signaling networks [22,56].

Building on prior research demonstrating the ability of *GPAT* genes to control abiotic stress responses, the transcriptional responsiveness of *GmGPAT* genes under stress conditions was evaluated through expression profiling. Differential expression patterns were observed in response to alkaline, saline, drought, and osmotic stress, with salt stress triggering the most pronounced transcriptional changes (Figure 7). Among these, GmGPAT1 exhibited the most rapid and robust upregulation following salt exposure, indicating its potential central role in mediating salt tolerance. Subcellular localization assays in *A. thaliana* protoplasts confirmed that GmGPAT1 localizes to the chloroplast compartment, consistent with computational predictions (Figure 6). *Agrobacterium*-mediated overexpression of *GmGPAT1* in transgenic plants afforded superior salt tolerance as compared to wild-type controls (Figure 9). The plants overexpressing *GmGPAT1* (*GmGPAT1*-OHR) presented with significantly reduced electrolyte leakage, consistent with the ability of *GmGPAT1* to mitigate damage to the membrane caused by salt stress through reductions in ROS levels and enhanced SOD, POD, CAT, APX, and GST activity (Figure 9). Whole-genome-sequencing-based candidate gene association analyses have increasingly been used to assess crop species in recent years [57,58], enabling the mining of beneficial alleles based on natural variability, narrowing the target range, and affording enhanced target gene identification. When analyzing *GmGPAT1* gene polymorphisms across soybean varieties to gain insight into how this gene shapes salt tolerance and its relationship to evolutionary history, a significant correlation was noted between natural *GmGPAT1* variation and salt tolerance. Two distinctly different haplotypes exhibited markedly divergent salt tolerance, with Hap-1 showing a frequency of 29%, thereby supporting the role of *GmGPAT1* as a key regulator of these stress responses. Together, these results provide new insights into the function of *GmGPAT1* in regulating salt tolerance while also shedding light on the evolutionary history of soybeans.

## 5. Conclusions

This study presents a comprehensive analysis of the *GmGPAT* gene family in the soybean, emphasizing the functional relevance of *GmGPAT1*, a chloroplast-localized isoform, in salinity stress adaptation. Twenty-seven *GmGPAT* genes were identified and classified into three phylogenetic and functional subgroups based on evolutionary relationships, domain architecture, and predicted subcellular localization. Expression analyses under various abiotic stresses revealed that many *GmGPAT* genes are preferentially expressed in the roots, with GmGPAT1 showing the strongest and most rapid induction under salt stress. A biochemical characterization of recombinant GmGPAT1 confirmed its function as an active GPAT enzyme, with a K_m_ of 36.5 mmol L^−1^ for G3P. Functional validation using transgenic hairy roots demonstrated that *GmGPAT1* overexpression confers enhanced salt tolerance by improving root architecture, boosting photosynthetic performance, and preserving membrane integrity under high-salt conditions. These phenotypes were tied to higher activity levels for ROS-scavenging enzymes (SOD, POD, CAT) and improved redox balance, as evidenced by higher AsA/DHA and GSH/GSSG ratios. Conversely, the *GmGPAT1* knockout lines were highly susceptible to salt stress, exhibiting elevated sodium levels, increased ROS accumulation, and extensive cell damage. A natural variation analysis identified a favorable promoter haplotype (*Hap-1*) associated with higher stress-tolerance indices among 288 soybean accessions, offering a promising molecular marker for breeding programs aimed at improving salt resilience. Together, these findings establish *GmGPAT1* as a critical integrator of glycerolipid metabolism, ion homeostasis, and oxidative stress responses under saline conditions. This work not only enriches the functional landscape of *GPAT* genes in the soybean but provides practical genetic tools and molecular insights for developing salt-tolerant soybean cultivars.

## Data Availability

All data generated and analyzed are included in this manuscript and its Supplementary Files.

## Supplementary Materials

The following supporting information can be downloaded at: https://www.mdpi.com/article/10.3390/plants14182862/s1. Figure S1: Identification of GmGPAT1 transgenic soybean hair roots. (**A**) PCR verification of GmGPAT1 in the 35s: GmGPAT1-overexpressing hairy roots (OHR1-10). M: DNA marker; Vector: the plasmid of empty vector (pSOY Ⅰ-GFP), as the negative control; GmGPAT1: the recombinant expression plasmid of pSOY Ⅰ-GmGPAT1/GFP, as the positive control. M: 2K PLUS. (**B**) The expression levels of GmGPAT1 in GmGPAT1-overexpressing hairy roots (OHR) compared with the control hairy roots (CHR). (**C**) Images were captured approximately 3 weeks post-transformation to assess GFP fluorescence using a Leica stereomicroscope; Figure S2: (**A**) After being treated with 120 mM NaCl for 5 days, CHR, GPAT-OHR, and GPAT-KHR were imaged using PlantExplorer PRO+. (**B**) After being treated with 120 mM NaCl for 5 days, the content of F0, Fm, Fv/Fm, Chl-index, and Ari-index of CHR, GPAT-OHR, and GPAT-KHR were measured. Double asterisks (**) and an asterisk (*) indicate significant difference at *p* < 0.01 and *p* < 0.05, shown by Student’s t-test; Table S1: Details of 86 identified GPAT genes; Table S2: The primers used in this study; Table S3: Collinear gene pairs of GPATs in five species; Table S4: Deduced protein sequences of GmGPAT1 in mutants compared to the WT.

## References

[1] Zou J., Wei Y., Jako C., Kumar A., Selvaraj G., Taylor D.C. (1999). The *Arabidopsis thaliana* TAG1 mutant has a mutation in a diacylglycerol acyltransferase gene. Plant J..

[2] Wendel A.A., Lewin T.M., Coleman R.A. (2009). Glycerol-3-phosphate acyltransferases: Rate limiting enzymes of triacylglycerol biosynthesis. Biochim. Biophys. Acta.

[3] Hurlock A.K., Roston R.L., Wang K., Benning C. (2014). Lipid trafficking in plant cells. Traffic.

[4] Zhou X.R., Bhandari S., Johnson B.S., Kotapati H.K., Allen D.K., Vanhercke T., Bates P.D. (2020). Reorganization of Acyl Flux through the Lipid Metabolic Network in Oil-Accumulating Tobacco Leaves. Plant Physiol..

[5] Zhang M., Fan J., Taylor D.C., Ohlrogge J.B. (2009). DGAT1 and PDAT1 acyltransferases have overlapping functions in *Arabidopsis* triacylglycerol biosynthesis and are essential for normal pollen and seed development. Plant Cell.

[6] Xu X., Yan B., Zhao Y., Wang F., Zhao X., He L., Xu J., Zhao C. (2019). Characterization and expression analysis of *GPAT* gene family in maize. Can. J. Plant Sci..

[7] Waschburger E., Kulcheski F.R., Veto N.M., Margis R., Margis-Pinheiro M., Turchetto-Zolet A.C. (2018). Genome-wide analysis of the Glycerol-3-Phosphate Acyltransferase (GPAT) gene family reveals the evolution and diversification of plant GPATs. Genet. Mol. Biol..

[8] Zhu T., Wu S., Zhang D., Li Z., Xie K., An X., Ma B., Hou Q., Dong Z., Tian Y. (2019). Genome-wide analysis of maize GPAT gene family and cytological characterization and breeding application of ZmMs33/ZmGPAT6 gene. Theor. Appl. Genet..

[9] Men X., Shi J., Liang W., Zhang Q., Lian G., Quan S., Zhu L., Luo Z., Chen M., Zhang D. (2017). Glycerol-3-Phosphate Acyltransferase 3 (OsGPAT3) is required for anther development and male fertility in rice. J. Exp. Bot..

[10] Zhou Y., Huang X., Hu T., Chen S., Wang Y., Shi X., Yin M., Li R., Wang J., Jia X. (2023). Genome-Wide Analysis of Glycerol-3-Phosphate Acyltransferase (GPAT) Family in *Perilla frutescens* and Functional Characterization of PfGPAT9 Crucial for Biosynthesis of Storage Oils Rich in High-Value Lipids. Int. J. Mol. Sci..

[11] Liu F., Xia Y., Wu L., Fu D., Hayward A., Luo J., Yan X., Xiong X., Fu P., Wu G. (2015). Enhanced seed oil content by overexpressing genes related to triacylglyceride synthesis. Gene.

[12] Payá-Milans M., Venegas-Calerón M., Salas J.J., Garcés R., Martínez-Force E. (2015). Cloning, heterologous expression and biochemical characterization of plastidial sn-glycerol-3-phosphate acyltransferase from *Helianthus annuus*. Phytochemistry.

[13] Sui N., Li M., Zhao S.J., Li F., Liang H., Meng Q.W. (2007). Overexpression of glycerol-3-phosphate acyltransferase gene improves chilling tolerance in tomato. Planta.

[14] Lv Y., Zhang X., Luo L., Yang H., Li P., Zhang K., Liu F., Wan Y. (2020). Characterization of glycerol-3-phosphate acyltransferase 9 (AhGPAT9) genes, their allelic polymorphism and association with oil content in peanut (*Arachis hypogaea* L.). Sci. Rep..

[15] Cui Y., Ma J., Liu G., Wang N., Pei W., Wu M., Li X., Zhang J., Yu J. (2019). Genome-Wide Identification, Sequence Variation, and Expression of the Glycerol-3-Phosphate Acyltransferase (GPAT) Gene Family in *Gossypium*. Front. Genet..

[16] Liu H., Wei L., Zhu J., Zhang B., Gan Y., Zheng Y. (2022). Identification of GmGPATs and their effect on glycerolipid biosynthesis through seed-specific expression in soybean. Mol. Biol. Rep..

[17] Muñoz C.F., Weusthuis R.A., D’Adamo S., Wijffels R.H. (2019). Effect of Single and Combined Expression of Lysophosphatidic Acid Acyltransferase, Glycerol-3-Phosphate Acyltransferase, and Diacylglycerol Acyltransferase on Lipid Accumulation and Composition in *Neochloris oleoabundans*. Front. Plant Sci..

[18] Li X.C., Zhu J., Yang J., Zhang G.R., Xing W.F., Zhang S., Yang Z.N. (2012). Glycerol-3-phosphate acyltransferase 6 (GPAT6) is important for tapetum development in *Arabidopsis* and plays multiple roles in plant fertility. Mol. Plant.

[19] Chen X., Chen G., Truksa M., Snyder C.L., Shah S., Weselake R.J. (2014). Glycerol-3-phosphate acyltransferase 4 is essential for the normal development of reproductive organs and the embryo in *Brassica napus*. J. Exp. Bot..

[20] Ouyang L.L., Li H., Yan X.J., Xu J.L., Zhou Z.G. (2016). Site-Directed Mutagenesis from Arg195 to His of a Microalgal Putatively Chloroplastidial Glycerol-3-Phosphate Acyltransferase Causes an Increase in Phospholipid Levels in Yeast. Front. Plant Sci..

[21] Zheng J., Yang J., Yang X., Cao Z., Cai S., Wang B., Ye J., Fu M., Zhang W., Rao S. (2022). Transcriptome and miRNA sequencing analyses reveal the regulatory mechanism of α-linolenic acid biosynthesis in *Paeonia rockii*. Food Res. Int..

[22] Sui N., Tian S., Wang W., Wang M., Fan H. (2017). Overexpression of Glycerol-3-Phosphate Acyltransferase from *Suaeda salsa* Improves Salt Tolerance in Arabidopsis. Front. Plant Sci..

[23] Sui N., Li M., Shu D.F., Zhao S.J., Meng Q.W. (2007). Antisense-mediated depletion of tomato chloroplast glycerol-3-phosphate acyltransferase affects male fertility and increases thermal tolerance. Physiol. Plant..

[24] Sun S.K., Yang N.N., Chen L.J., Irfan M., Zhao X.H., Li T.L. (2015). Characterization of LpGPAT gene in *Lilium pensylvanicum* and response to cold stress. Biomed Res. Int..

[25] Wu F., Chen Z., Zhang F., Zheng H., Li S., Gao Y., Yang J., Sui N. (2022). Identification and Transcriptome Analysis of Genes Related to Membrane Lipid Regulation in Sweet Sorghum under Salt Stress. Int. J. Mol. Sci..

[26] Yu C.S., Lin C.J., Hwang J.K. (2004). Predicting subcellular localization of proteins for Gram-negative bacteria by support vector machines based on n-peptide compositions. Protein Sci..

[27] Tamura K., Peterson D., Peterson N., Stecher G., Nei M., Kumar S. (2011). MEGA5: Molecular evolutionary genetics analysis using maximum likelihood, evolutionary distance, and maximum parsimony methods. Mol. Biol. Evol..

[28] Lee T.H., Tang H., Wang X., Paterson A.H. (2013). PGDD: A database of gene and genome duplication in plants. Nucleic Acids Res..

[29] Bailey T.L., Johnson J., Grant C.E., Noble W.S. (2015). The MEME Suite. Nucleic Acids Res..

[30] Liu W., Xie Y., Ma J., Luo X., Nie P., Zuo Z., Lahrmann U., Zhao Q., Zheng Y., Zhao Y. (2015). IBS: An illustrator for the presentation and visualization of biological sequences. Bioinformatics.

[31] Yoo S.D., Cho Y.H., Sheen J. (2007). *Arabidopsis* mesophyll protoplasts: A versatile cell system for transient gene expression analysis. Nat. Protoc..

[32] Bustin S.A., Benes V., Garson J.A., Hellemans J., Huggett J., Kubista M., Kubista M., Mueller R., Nolan T., Pfaffl M.W. (2009). The MIQE guidelines: Minimum information for publication of quantitative real-time PCR experiments. Clin. Chem..

[33] Zhao Y., Cao P., Cui Y., Liu D., Li J., Zhao Y., Yang S., Zhang B., Zhou R., Sun M. (2021). Enhanced production of seed oil with improved fatty acid composition by overexpressing NAD(+) -dependent glycerol-3-phosphate dehydrogenase in soybean. J. Integr. Plant Biol..

[34] Tóth K., Batek J., Stacey G. (2016). Generation of soybean (*Glycine max*) transient transgenic roots. Curr. Protoc. Plant Biol..

[35] Fryer M.J., Oxborough K., Mullineaux P.M., Baker N.R. (2002). Imaging of photo-oxidative stress responses in leaves. J. Exp. Bot..

[36] Velikova V., Yordanov I., Edreva A. (2000). Oxidative stress and some antioxidant systems in acid rain-treated bean plants: Protective role of exogenous polyamines. Plant Sci..

[37] Giannopolitis C.N., Ries S.K. (1977). Superoxide dismutase. I. occurrence in higher plants. J. Plant Physiol..

[38] Nakano Y., Asada K. (1981). Hydrogen peroxide is scavenged by ascorbate-specific peroxidase in spinach chloroplasts. Plant Cell Physiol..

[39] Zhang R., Hussain S., Wang Y., Liu Y., Li Q., Chen Y., Wei H., Gao P., Dai Q. (2021). Comprehensive Evaluation of Salt Tolerance in Rice (*Oryza sativa* L.) Germplasm at the Germination Stage. Agronomy.

[40] Han R., Lu X., Gao G. (2006). Analysis of the Principal Components and the Subordinate Function of Alfalfa Drought Resistance. Acta Agrestia Sin..

[41] Dai H., Wu H., Amanguli M., Wang L., Maimaiti A., Zhang J. (2014). Analysis of Salt-Tolerance and Determination of Salt-Tolerant Evaluation Indicators in Cotton Seedlings of Different Genotypes. Sci. Agric. Sin..

[42] Librado P., Rozas J. (2009). DnaSP v5: A Software for Comprehensive Analysis of DNA Polymorphism Data. Bioinformatics.

[43] Berkemeyer M., Scheibe R., Ocheretina O. (1998). A novel, non-redox-regulated NAD-dependent malate dehydrogenase from chloroplasts of *Arabidopsis thaliana* L.. J. Biol. Chem..

[44] Selinski J., König N., Wellmeyer B., Hanke G.T., Linke V., Neuhaus H.E., Scheibe R. (2014). The plastid-localized NAD-dependent malate dehydrogenase is crucial for energy homeostasis in developing *Arabidopsis thaliana* seeds. Mol. Plant.

[45] Sew Y.S., Ströher E., Fenske R., Millar A.H. (2016). Loss of mitochondrial malate dehydrogenase activity alters seed metabolism lmpairing seed maturation and post-germination growth in *Arabidopsis*. Plant Physiol..

[46] Wang Q.J., Sun H., Dong Q.L., Sun T.Y., Jin Z.X., Hao Y.J., Yao Y.X. (2016). The enhancement of tolerance to salt and cold stresses by modifying the redox state and salicylic acid content via the cytosolic malate dehydrogenase gene in transgenic apple plants. Plant Biotechnol. J..

[47] Qi J., Song C.P., Wang B., Zhou J., Kangasjärvi J., Zhu J.K., Hao Y.J., Yao Y.X. (2018). Reactive oxygen species signaling and stomatal movement in plant responses to drought stress and pathogen attack. J. Integr. Plant Biol..

[48] Bournonville C.F., Díaz-Ricci J.C. (2011). Quantitative determination of superoxide in plant leaves using a modified NBT staining method. Phytochem. Anal. PCA.

[49] Yan L.Y., Zhang H.J., Zheng Y.Q., Cong Y.Q., Liu C.T., Fan F., Zheng C., Yuan G.L., Pan G., Yuan D.Y. (2021). Transcription factor OsMADS25 improves rice tolerance to cold stress. Yi Chuan.

[50] Vijayaraghavareddy P., Adhinarayanreddy V., Vemanna R.S., Sreeman S., Makarla U. (2017). Quantification of Membrane Damage/Cell Death Using Evan’s Blue Staining Technique. Bio-Protoc..

[51] Cao D., Li Y., Liu B., Kong F., Tran L.S.P. (2018). Adaptive mechanisms of soybean grown on salt-affected soils. Land Degrad. Dev..

[52] Jia Q., Bai Y., Xu H., Liu Q., Li W., Li T., Lin F., Shen L., Xuan W., Zhang W. (2022). Mitochondrial GPAT-derived LPA controls auxin-dependent embryonic and postembryonic development. Proc. Natl. Acad Sci. USA.

[53] Payá-Milans M., Aznar-Moreno J.A., Balbuena T.S., Haslam R.P., Gidda S.K., Pérez-Hormaeche J., Mullen R.T., Thelen J.J., Napier J.A., Salas J.J. (2016). Sunflower HaGPAT9-1 is the predominant GPAT during seed development. Plant Sci..

[54] Yang C., Ma J., Qi C., Ma Y., Xiong H., Duan R. (2024). Genome-Wide Identification, Characterization, Evolutionary Analysis, and Expression Pattern of the GPAT Gene Family in Barley and Functional Analysis of HvGPAT18 under Abiotic Stress. Int. J. Mol. Sci..

[55] Zhang X., Gao H., Liu Y., Zhao H., Lü S. (2024). Function identification of Arabidopsis GPAT4 and GPAT8 in the biosynthesis of suberin and cuticular wax. Plant Sci..

[56] Tamada T., Feese M.D., Ferri S.R., Kato Y., Yajima R., Toguri T., Kuroki R. (2004). Substrate recognition and selectivity of plant glycerol-3-phosphate acyltransferases (GPATs) from Cucurbita moscata and Spinacea oleracea. Acta Crystallogr. D Biol. Crystallogr..

[57] Zhu G., Gao W., Song X., Sun F., Hou S., Liu N., Huang Y., Zhang D., Ni Z., Chen Q. (2020). Genome-wide association reveals genetic variation of lint yield components under salty field conditions in cotton (*Gossypium hirsutum* L.). BMC Plant Biol..

[58] Chu S., Zhang X., Yu K., Lv L., Sun C., Liu X., Zhang J., Jiao Y., Zhang D. (2020). Genome-Wide Analysis Reveals Dynamic Epigenomic Differences in Soybean Response to Low-Phosphorus Stress. Int. J. Mol. Sci..

