# Supplementary material for: Characterization of the Soybean GPAT Gene Family Identifies GmGPAT1 as a Key Protein in Salt Stress Tolerance
Source: Plants (Basel). 2025 Sep 13;14(18):2862. doi: 10.3390/plants14182862 (PMC12473205; doi:10.3390/plants14182862)
Supplement: Supplementary file 1 [file plants-14-02862-s001.zip › Supplementary Figures.pdf]

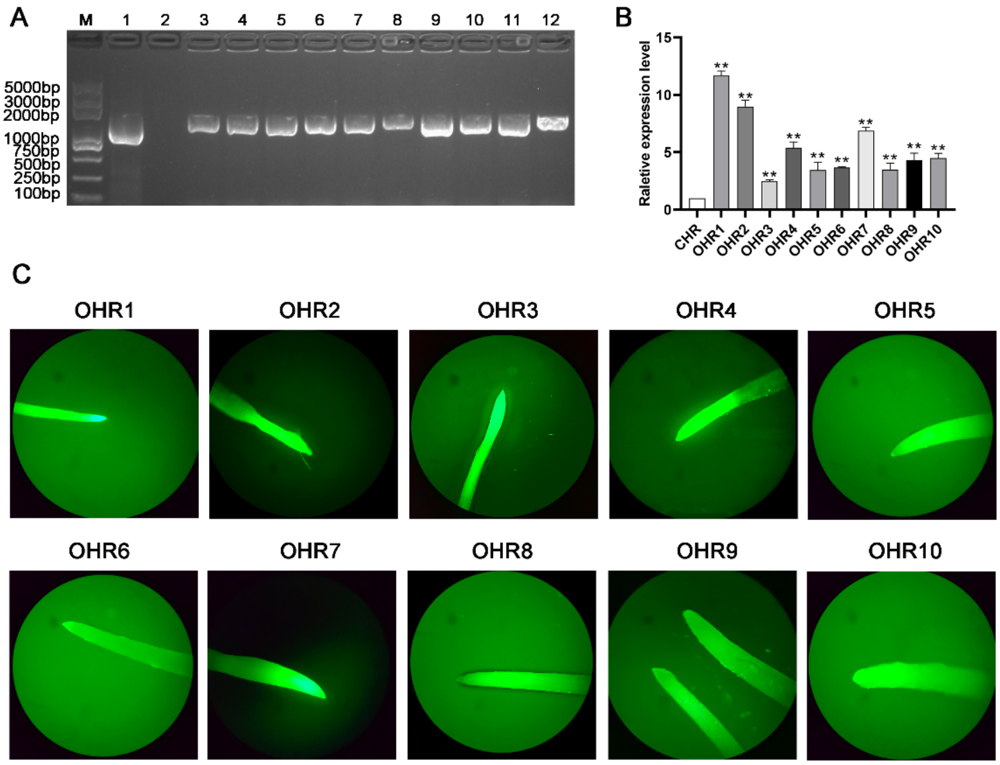

Figure. S1 Identification of GmGPAT1 transgenic soybean hair roots. (A) PCR verification of GmGPAT1 in the 35s: GmGPAT1-overexpressing hairy roots (OHR1-10). M: DNA marker; Vector: the plasmid of empty vector (pSOY I-GFP), as the negative control; GmGPAT1: the recombinant expression plasmid of pSOY I-GmGPAT1:GFP, as the positive control. M: 2K PLUS. (B) The expression levels of *GmGPAT1* in *GmGPAT1*-overexpressing hairy roots (OHR) compared with the control hairy roots (CHR). (C) Images were captured approximately 3 weeks post-transformation to assess GFP fluorescence using a Leica stereomicroscope

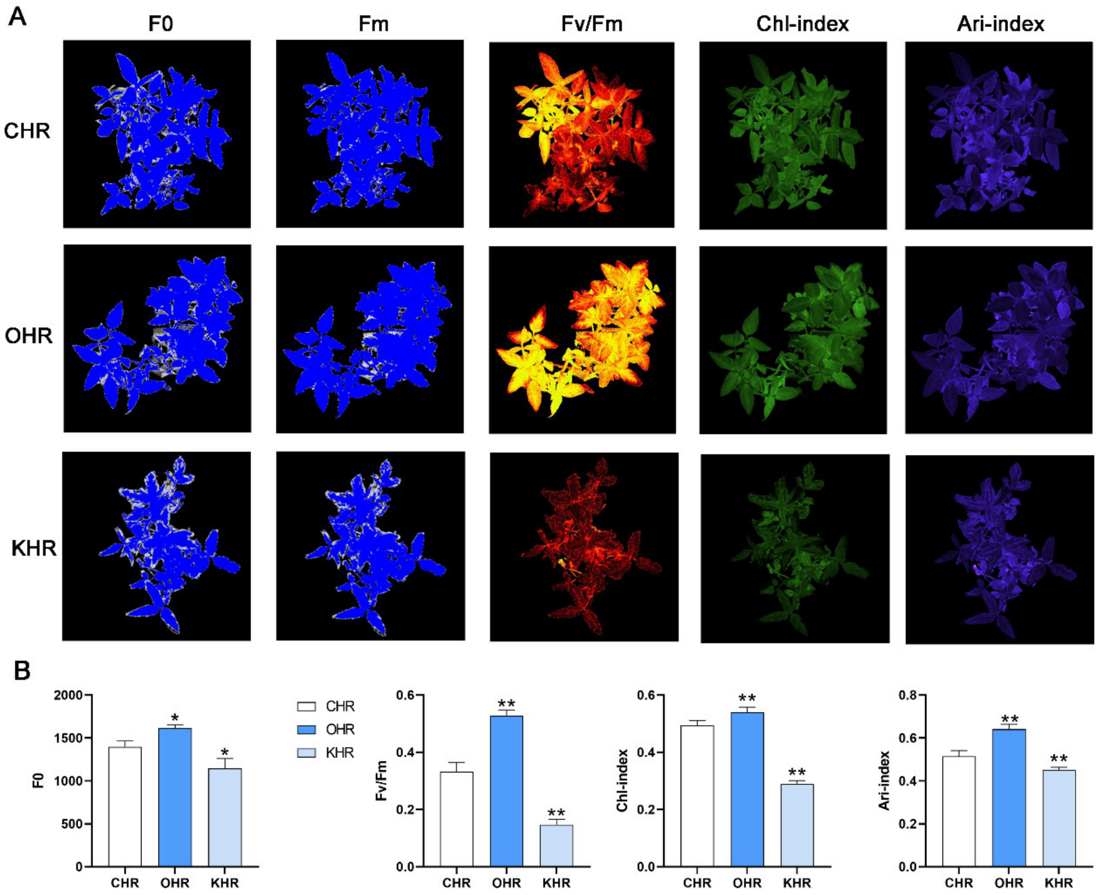

Figure. S2 (A) After being treated with 120 mM NaCl for 5 days, CHR, GPAT-OHR, and GPAT-KHR were imaged using PlantExplorer PRO+. (B) After being treated with 120 mM NaCl for 5 days, the content of F0, Fm, Fv/Fm, Chl-index and Ari-index of CHR, GPAT-OHR, and GPAT-KHR were measured. Double asterisks (∗∗) and an asterisk (∗) indicate significant difference at $P < 0.01$ and $P < 0.05$, shown by Student's *t*-test.
