# Supplementary material for: Characterization of the Soybean GPAT Gene Family Identifies GmGPAT1 as a Key Protein in Salt Stress Tolerance
Source: Plants (Basel). 2025 Sep 13;14(18):2862. doi: 10.3390/plants14182862 (PMC12473205; doi:10.3390/plants14182862)
Supplement: Supplementary file 1 [file plants-14-02862-s001.zip › Table S1.pdf]

Table S1 – Details of 86 identified *GPAT* genes.

Name	Gene ID	Gene location	CDS (bp)	Protein length	PI	MW (kDa)	Subcellular localization
GmGPAT1	*Glyma.01G014200*	Chr01 1371902..1382029	1377	459	8.36	50.48	Chloroplast
GmGPAT2	*Glyma.01G113200*	Chr01 38755064..38758956	1479	493	9.46	54.38	Mitochondrion
GmGPAT3	*Glyma.02G010600*	Chr02 1006941..1009796	1668	556	9.67	63.07	Mitochondrion
GmGPAT4	*Glyma.02G249300*	Chr02 43682558..43685068	1419	473	9.69	52.82	Endoplasmic reticulum
GmGPAT5	*Glyma.02G286500*	Chr02 46721797..46724307	1620	540	8.58	60.19	Endoplasmic reticulum
GmGPAT6	*Glyma.03G008300*	Chr03 801854..806650	1503	501	9.66	55.73	Mitochondrion
GmGPAT7	*Glyma.03G078600*	Chr03 19691949..19696306	1497	499	9.78	55.22	Mitochondrion
GmGPAT8	*Glyma.03G221100*	Chr03 42427489..42430622	1620	540	9.54	61.32	Mitochondrion
GmGPAT9	*Glyma.03G221300*	Chr03 42439999..42443047	1530	510	9.21	57.22	Mitochondrion
GmGPAT10	*Glyma.05G131100*	Chr05 32411640..32419257	1122	374	8.99	42.84	Endoplasmic reticulum
GmGPAT11	*Glyma.07G069700*	Chr07 6316519..6321126	1500	500	9.66	55.53	Mitochondrion
GmGPAT12	*Glyma.07G146800*	Chr07 17588033..17591814	1491	497	9.32	55.06	Mitochondrion
GmGPAT13	*Glyma.08G085800*	Chr08 6465894..6472527	1122	374	8.99	42.82	Chloroplast
GmGPAT14	*Glyma.08G309200*	Chr08 42818068..42821065	1602	534	8.94	60.65	Mitochondrion
GmGPAT15	*Glyma.09G119200*	Chr09 28544564..28555723	1131	377	9.2	43.37	Endoplasmic reticulum
GmGPAT16	*Glyma.09G207900*	Chr09 43215178..43225630	1413	471	8.62	51.81	Chloroplast
GmGPAT17	*Glyma.10G011000*	Chr10 1040838..1043359	1671	557	9.81	63.20	Mitochondrion
GmGPAT18	*Glyma.10G119900*	Chr10 30516479..30519657	1530	510	9.63	55.59	Mitochondrion
GmGPAT19	*Glyma.13G085700*	Chr13 19760151..19762223	1512	504	8.92	56.50	Mitochondrion
GmGPAT20	*Glyma.14G028300*	Chr14 2056434..2059029	1623	541	8.70	60.33	Mitochondrion
GmGPAT21	*Glyma.14G067200*	Chr14 5567989..5570401	1419	473	9.57	53.16	Mitochondrion
GmGPAT22	*Glyma.14G167300*	Chr14 41382511..41384467	1518	506	9.12	56.95	Mitochondrion
GmGPAT23	*Glyma.14G167400*	Chr14 41489761..41492199	1494	498	8.33	56.23	Mitochondrion
GmGPAT24	*Glyma.18G107100*	Chr18 12047166..12050021	1584	528	8.55	60.00	Mitochondrion
GmGPAT25	*Glyma.18G197800*	Chr18 47499401..47503080	1620	540	9.41	59.48	Mitochondrion
GmGPAT26	*Glyma.19G218100*	Chr19 47082132..47085750	1623	541	9.33	61.38	Mitochondrion
GmGPAT27	*Glyma.20G070400*	Chr20 25080767..25083537	1539	513	9.62	55.97	Mitochondrion
AtGPAT1	*AT1G01610*	Chr1 221690..224340	1512	504	9.48	56.27	Mitochondrion
AtGPAT2	*AT1G02390*	Chr1 480851..483292	1593	531	9.78	59.98	Mitochondrion
AtGPAT3	*AT1G06520*	Chr1 1993977..1996112	1758	586	9.47	66.52	Mitochondrion

Name	Gene ID	Gene location	CDS (bp)	Protein length	PI	MW (kDa)	Subcellular localization
AtGPAT4	*AT1G32200*	Chr1 11601780..11605060	1380	460	6.52	50.43	Chloroplast
AtGPAT5	*AT2G38110*	Chr2 15952606..15955494	1506	502	10.02	56.14	Mitochondrion
AtGPAT6	*AT3G11430*	Chr3 3595764..3597952	1509	503	9.52	56.09	Mitochondrion
AtGPAT7	*AT4G00400*	Chr4 174061..176853	1503	501	9.31	55.87	Mitochondrion
AtGPAT8	*AT4G01950*	Chr4 844408..846787	1563	521	9.70	58.77	Mitochondrion
AtGPAT9	*AT5G06090*	Chr5 1835105..1836722	1503	501	8.72	56.09	Mitochondrion
AtGPAT10	*AT5G60620*	Chr5 24367197..24369790	1131	377	8.67	43.03	Endoplasmic reticulum
OsGPAT1	*LOC_Os01g14900*	Chr1 8347892..8351006	1713	571	8.90	60.31	Mitochondrion
OsGPAT2	*LOC_Os01g19390*	Chr1 10969330..10972881	1362	454	8.77	51.25	Mitochondrion
OsGPAT3	*LOC_Os01g22560*	Chr1 12677085..12678141	795	265	9.20	28.03	Chloroplast
OsGPAT4	*LOC_Os01g44069*	Chr1 25257000..25263981	1635	545	9.89	59.69	Mitochondrion
OsGPAT5	*LOC_Os01g63580*	Chr1 36863213..36866375	1494	498	9.72	55.69	Mitochondrion
OsGPAT6	*LOC_Os02g02340*	Chr2 775493..778356	1521	507	9.91	54.56	Mitochondrion
OsGPAT7	*LOC_Os02g24340*	Chr2 14121102..14123910	831	277	8.08	31.09	Mitochondrion
OsGPAT8	*LOC_Os03g52570*	Chr3 30151747..30153564	1404	468	9.10	50.61	Mitochondrion
OsGPAT9	*LOC_Os03g61720*	Chr3 34985195..34990759	1626	542	9.67	58.89	Mitochondrion
OsGPAT10	*LOC_Os05g20100*	Chr5 11753280..11756381	1614	538	9.50	59.79	Mitochondrion
OsGPAT11	*LOC_Os05g37600*	Chr5 22005195..22008976	1461	487	9.14	53.08	Mitochondrion
OsGPAT12	*LOC_Os07g34730*	Chr7 20808032..20812544	1113	371	8.55	42.63	Endoplasmic reticulum
OsGPAT13	*LOC_Os08g03700*	Chr8 1757710..1759198	1488	496	9.35	52.48	Mitochondrion
OsGPAT14	*LOC_Os10g27330*	Chr10 14424221..14428197	1593	531	8.88	56.66	Mitochondrion
OsGPAT15	*LOC_Os10g41070*	Chr10 22059228..22060717	1440	480	10.80	51.22	Mitochondrion
OsGPAT16	*LOC_Os10g42720*	Chr10 23044692..23050740	1284	428	6.47	47.87	Chloroplast
OsGPAT17	*LOC_Os11g45400*	Chr11 27486902..27490269	1632	544	9.19	60.12	Mitochondrion
OsGPAT18	*LOC_Os12g37600*	Chr12 23091635..23094141	1677	559	9.90	59.80	Mitochondrion
SbGPAT1	*Sobic.001G026100*	Chr01 2030755..2033997	1710	570	9.82	60.50	Mitochondrion
SbGPAT2	*Sobic.001G099300*	Chr01 7598407..7600784	1518	506	9.01	55.13	Mitochondrion
SbGPAT3	*Sobic.001G250200*	Chr01 26998412..27003143	1557	519	9.11	55.84	Mitochondrion
SbGPAT4	*Sobic.001G283700*	Chr01 55609080..55626018	1347	449	6.96	49.80	Chloroplast
SbGPAT5	*Sobic.001G493300*	Chr01 76240810..76244345	1575	525	8.68	56.67	Mitochondrion
SbGPAT6	*Sobic.002G325300*	Chr02 69565614..69571744	1116	372	8.71	42.84	Endoplasmic reticulum
SbGPAT7	*Sobic.003G142500*	Chr03 14157136..14160771	1614	538	9.07	61.72	Endoplasmic reticulum

Name	Gene ID	Gene location	CDS (bp)	Protein length	PI	MW (kDa)	Subcellular localization
SbGPAT8	*Sobic.003G229700*	Chr03 56945924..56952742	1719	573	9.85	62.96	Mitochondrion
SbGPAT9	*Sobic.003G360700*	Chr03 67827782..67831882	1512	504	9.68	56.19	Mitochondrion
SbGPAT10	*Sobic.004G010300*	Chr04 866842..869960	1557	519	9.64	55.77	Mitochondrion
SbGPAT11	*Sobic.005G214400*	Chr05 70061853..70064960	1746	582	9.50	62.71	Endoplasmic reticulum
SbGPAT12	*Sobic.008G130800*	Chr08 55678677..55680679	1812	604	9.59	64.05	Endoplasmic reticulum
SbGPAT13	*Sobic.009G157000*	Chr09 51459015..51462032	1533	511	9.69	55.85	Mitochondrion
SbGPAT14	*Sobic.009G202600*	Chr09 55163707..55165354	1494	498	10.17	53.21	Mitochondrion
ZmGPAT1	*Zm00001eb005110_T001*	Chr01 14142523..14145101	1521	507	9.51	54.82	Mitochondrion
ZmGPAT2	*Zm00001eb025080_T001*	Chr01 106169616..106177031	1344	448	7.18	49.98	Chloroplast
ZmGPAT3	*Zm00001eb048150_T001*	Chr01 247180167..247187376	1566	522	8.77	56.22	Chloroplast
ZmGPAT4	*Zm00001eb063420_T001*	Chr01 302404545..302407061	1614	538	9.94	57.33	Endoplasmic reticulum
ZmGPAT5	*Zm00001eb115970_T001*	Chr02 237369704..237371991	1578	526	10.12	57.13	Mitochondrion
ZmGPAT6	*Zm00001eb127520_T001*	Chr03 35530277..35533946	1629	543	9.69	58.89	Mitochondrion
ZmGPAT7	*Zm00001eb136910_T001*	Chr03 130143796..130147128	1788	596	9.70	63.60	Endoplasmic reticulum
ZmGPAT8	*Zm00001eb147550_T001*	Chr03 183207240..183210354	1509	503	9.48	55.94	Mitochondrion
ZmGPAT9	*Zm00001eb159140_T001*	Chr03 222010236..222016466	1638	546	9.84	60.13	Mitochondrion
ZmGPAT10	*Zm00001eb173160_T001*	Chr04 38063626..38065642	1491	497	9.28	52.75	Mitochondrion
ZmGPAT11	*Zm00001eb224960_T002*	Chr05 46523224..46536618	642	214	4.57	24.54	Mitochondrial
ZmGPAT12	*Zm00001eb229800_T001*	Chr05 71113706..71115765	1548	516	9.44	54.31	Mitochondrial
ZmGPAT13	*Zm00001eb323170_T002*	Chr07 163430145..163442564	1116	372	8.87	42.86	Endoplasmic reticulum
ZmGPAT14	*Zm00001eb347840_T001*	Chr08 98428198..9843075	1461	487	8.80	52.96	Mitochondrion
ZmGPAT15	*Zm00001eb351250_T002*	Chr08 118777861..118780292	1497	499	9.61	54.03	Mitochondrion
ZmGPAT16	*Zm00001eb358940_T001*	Chr08 149786377..149794218	1674	558	10.19	61.21	Mitochondrion
ZmGPAT17	*Zm00001eb396350_T003*	Chr09 142156843..142162927	1116	372	8.71	42.76	Endoplasmic reticulum
