# Supplementary material for: Characterization of the Soybean GPAT Gene Family Identifies GmGPAT1 as a Key Protein in Salt Stress Tolerance
Source: Plants (Basel). 2025 Sep 13;14(18):2862. doi: 10.3390/plants14182862 (PMC12473205; doi:10.3390/plants14182862)
Supplement: Supplementary file 1 [file plants-14-02862-s001.zip › Table S2.pdf]

Table.S2 The primers used in this study.

Primer Names	Sequence (5'-3')	Description
GmGPAT1-F	ATGAGCACGACCGGTTCTTC	cloning
GmGPAT1-R	CTTCCATGACTGTGACAAAG	cloning
GmGPAT3-F	ATGGCTAAATTGTTCAGAGC	cloning
GmGPAT3-R	AGAGTTGGACTTGCTTATGC	cloning
GmGPAT4-F	ATGGAGTCTGTGGTGTGTGA	cloning
GmGPAT4-R	CCGTATGAACGTTCTCACCA	cloning
pET28a(+)-GPAT1-F	ATGAGCACGACCGGTTCTTC	cloning
pET28a(+)-GPAT1-R	CTTCCATGACTGTGACAAAG	cloning
pSOY1-GPAT2-F	CATTCAGTATTGGCAGGAAT	PCR verification
pSOY1-GFP-R	TTGTACAGCTCGTCCATGCC	PCR verification
GmGPAT1-qF	CGGTTCTTCGGCTTACCACT	qRT-PCR
GmGPAT1-qR	AAACGGAAGAACCCTAGGCG	qRT-PCR
GmGPAT2-qF	CGATAGGATTGTCCCGGTCG	qRT-PCR
GmGPAT2-qR	CCCATGCAGGTGAGTTCTGT	qRT-PCR
GmGPAT3-qF	GGACCTAGTTGTGTGTCCGG	qRT-PCR
GmGPAT3-qR	GCGGGGTTCATGAGGAAGAA	qRT-PCR
GmGPAT4-qF	GCTGAACTAACCGACCGGAT	qRT-PCR
GmGPAT4-qR	CCCCGAAGAACACGTAGCTT	qRT-PCR
GmGPAT5-qF	CCTGAGGCTTGCAGATTGGA	qRT-PCR
GmGPAT5-qR	CTGGGGAAAGAGGAAGGCTG	qRT-PCR
GmGPAT6-qF	ACGGACCGTGACTTCATGTC	qRT-PCR
GmGPAT6-qR	AGGGCATTCATTGGGTCTGG	qRT-PCR
GmGPAT7-qF	AGAGGGACAAAGATGCTGCC	qRT-PCR
GmGPAT7-qR	TTTGCTTGGTGTTGATGGCG	qRT-PCR
GmGPAT8-qF	GAGCTGGTTCGTGAAGGGAA	qRT-PCR
GmGPAT8-qR	CTTCTCAGCTTCCGTCACCA	qRT-PCR
GmGPAT9-qF	ATTGCTCAAGGGGCGTCTTT	qRT-PCR

GmGPAT9-qR	GTTCCTCGTTAAACGCACCG	qRT-PCR
GmGPAT10-qF	TTTGAAGCCAGGGGAGACAC	qRT-PCR
GmGPAT10-qR	CAGCACAGACTCAGCGAAGA	qRT-PCR
GmGPAT11-qF	TTCCCGCCTATCACCGAATG	qRT-PCR
GmGPAT11-qR	TAGGCGATGATGACGAACGG	qRT-PCR
GmGPAT12-qF	ATTGAGATGGTCGCGTCCTC	qRT-PCR
GmGPAT12-qR	CAAGCACCTTATCAGCCCCA	qRT-PCR
GmGPAT13-qF	CTCACATCGTGCTGGGCTTA	qRT-PCR
GmGPAT13-qR	ATTTTTCCTCAAAGCGCCGC	qRT-PCR
GmGPAT14-qF	CTGGCTGTTACTTCACGGGT	qRT-PCR
GmGPAT14-qR	TACCGATCCCAAGGTCAGGT	qRT-PCR
GmGPAT15-qF	CTCAGTTCGTGCTGGCCTTA	qRT-PCR
GmGPAT15-qR	TCACTTTTCCTCCCATCGCC	qRT-PCR
GmGPAT16-qF	CTCCTCCTCCATCACTCCCA	qRT-PCR
GmGPAT16-qR	TCGTTCTTCGCGTTGAGGAA	qRT-PCR
GmGPAT17-qF	TTTCTCATGAACCCCGAGCC	qRT-PCR
GmGPAT17-qR	ACGCGGTACCAATCTGTGTT	qRT-PCR
GmGPAT18-qF	ACTGATGCTCCCTTCATGGC	qRT-PCR
GmGPAT18-qR	AAAGAATGGTGAGGAGGGCG	qRT-PCR
GmGPAT19-qF	GCTTTGAGGCCAACTCTTGC	qRT-PCR
GmGPAT19-qR	TTCTTGTTCCTGTGAGGGCC	qRT-PCR
GmGPAT20-qF	TGGTTCTGGCTTGCTTGTGA	qRT-PCR
GmGPAT20-qR	TGGCAACACTGAACTTGGGT	qRT-PCR
GmGPAT21-qF	ACATGTCGCGAACCCTTTCT	qRT-PCR
GmGPAT21-qR	TAACCTCGTACACCGGCCTA	qRT-PCR
GmGPAT22-qF	AGCTGGTGGGGTACTACGAT	qRT-PCR
GmGPAT22-qR	GCCTCAAAGCCTTCCAATCC	qRT-PCR
GmGPAT23-qF	TCATGAACCCGAATCCCACG	qRT-PCR
GmGPAT23-qR	TTGACACCCTCGTTACCTGC	qRT-PCR

GmGPAT24-qF	AAGCCTGACCTTGGGATTGG	qRT-PCR
GmGPAT24-qR	AAGAAGGGTTGCGGAAGGAG	qRT-PCR
GmGPAT25-qF	CACTTCGAACCGGTCTCCAA	qRT-PCR
GmGPAT25-qR	GTACACGAAAGGGACGGAGG	qRT-PCR
GmGPAT26-qF	CACCAATCAAAACGGTGCGT	qRT-PCR
GmGPAT26-qR	TGGGACCATTTCATCGCACA	qRT-PCR
GmGPAT27-qF	AAGCCGGGGATACTTGTTGG	qRT-PCR
GmGPAT27-qR	GCCATGAAGGGAGCATCTGT	qRT-PCR
GmACTIN4-qF	GTGTCAGCCATACTGTCCCCATTT	qRT-PCR
GmACTIN4-qR	GTTTCAAGCTCTTGCTCGTAATCA	qRT-PCR
sgRNA1	CACGCACCTTCCTCAACGCTCGG	Specific gRNAs targeting *GmDAGT1s*
SgRNA2	GATGGAGGAGCAACAAGGCGAGG	
GmGPAT1-F1	GTTTCTCTCTTCAAAACT	Detection mutations of *GmGPAT1* target
GmGPAT1-R1	CGGACATGTTAGCGGGAGAA	
GmGPAT1-F2	TTCTCCCGCTAACATGTCCG	
GmGPAT1-R2	TGCACTTTTATAGTTATTGT	
